# Strand-specific RNA-seq reveals widespread occurrence of novel *cis-*natural antisense transcripts in rice

**DOI:** 10.1186/1471-2164-13-721

**Published:** 2012-12-22

**Authors:** Tingting Lu, Chuanrang Zhu, Guojun Lu, Yunli Guo, Yan Zhou, Zhiyong Zhang, Yan Zhao, Wenjun Li, Ying Lu, Weihua Tang, Qi Feng, Bin Han

**Affiliations:** 1 National Center for Gene Research & Institute of Plant Physiology and Ecology, Shanghai Institutes of Biological Sciences, Chinese Academy of Sciences, Shanghai, 200233, China; 2 National Key Laboratory of Plant Molecular Genetics, Institute of Plant Physiology and Ecology, Shanghai Institutes for Biological Sciences, Chinese Academy of Sciences, Shanghai, 200233, China

**Keywords:** Oryza sativa, *Cis-*NATs, Nat-siRNAs, SsRNA-seq, Transcriptome

## Abstract

**Background:**

*Cis-*natural antisense transcripts (*cis-*NATs) are RNAs transcribed from the antisense strand of a gene locus, and are complementary to the RNA transcribed from the sense strand. Common techniques including microarray approach and analysis of transcriptome databases are the major ways to globally identify *cis-*NATs in various eukaryotic organisms. Genome-wide *in silico* analysis has identified a large number of *cis-*NATs that may generate endogenous short interfering RNAs (nat-siRNAs), which participate in important biogenesis mechanisms for transcriptional and post-transcriptional regulation in rice. However, the transcriptomes are yet to be deeply sequenced to comprehensively investigate *cis-*NATs.

**Results:**

We applied high-throughput strand-specific complementary DNA sequencing technology (ssRNA-seq) to deeply sequence mRNA for assessing sense and antisense transcripts that were derived under salt, drought and cold stresses, and normal conditions, in the model plant rice (*Oryza sativa*). Combined with RAP-DB genome annotation (the Rice Annotation Project Database build-5 data set), 76,013 transcripts corresponding to 45,844 unique gene loci were assembled, in which 4873 gene loci were newly identified. Of 3819 putative rice *cis-*NATs, 2292 were detected as expressed and giving rise to small RNAs from their overlapping regions through integrated analysis of ssRNA-seq data and small RNA data. Among them, 503 *cis-*NATs seemed to be associated with specific conditions. The deep sequence data from isolated epidermal cells of rice seedlings further showed that 54.0% of *cis-*NATs were expressed simultaneously in a population of homogenous cells. Nearly 9.7% of rice transcripts were involved in one-to-one or many-to-many *cis-*NATs formation. Furthermore, only 17.4-34.7% of 223 many-to-many *cis-*NAT groups were all expressed and generated nat-siRNAs, indicating that only some *cis-*NAT groups may be involved in complex regulatory networks.

**Conclusions:**

Our study profiles an abundance of *cis-*NATs and nat-siRNAs in rice. These data are valuable for gaining insight into the complex function of the rice transcriptome.

## Background

*Cis-*natural antisense transcripts (*cis-*NATs) are endogenous RNA molecules that are transcribed from the opposite DNA strand of the same genomic locus and overlap partly with sense RNA by convergent or divergent orientation. It has been clearly demonstrated that *cis-*NATs are an important biogenesis mechanism to generate endogenous short interfering RNAs (siRNAs) known as ‘natural antisense siRNAs’ (nat-siRNAs) [1,2]. Numerous evidence indicates that NATs use diverse transcriptional and post-transcriptional gene regulatory mechanisms to carry out different biological roles [3], including RNA interference [4], gene silencing [5-7], RNA masking-induced alternative splicing [8] and RNA editing [9].

Since a large number of natural antisense transcripts were first identified to be widespread in the human genome [10], computational analysis of data generated from large-scale sequencing projects has been widely used to globally identify *cis-*NATs in various eukaryotic organisms. Common techniques and databases, including *in silico* analyses of expressed sequence tag (EST) databases [11,12], genomic annotation of large transcript sets [13-17], large-scale sequencing of full-length complementary DNAs (cDNAs) [18-20] and tiling arrays [21-24], have been applied to identify NATs. Recently, more efforts were made to characterize nat-siRNAs and NATs at a genome-wide level: massively parallel signature sequencing (MPSS) data [25,26], combining pyrophosphate-based high-throughput sequencing and computational analyses of genomic annotation datasets [27,28] and asymmetric strand-specific analysis of gene expression (ASSAGE) [29]. It has been reported that about 5-70% in mammals and 7-9% in plants of all transcripts are overlapped as *cis-*NATs. These studies have demonstrated four major characteristics of *cis-*NATs. (i) Both *cis-*NAT pairs can encode proteins or be non-protein-coding transcripts. In the mammalian genome, a non-protein-coding antisense RNA partner of a protein-coding transcript is considered the most prominent form [13,15]. In *Arabidopsis*, ~88% of the sense-antisense transcripts (6858 of 7805) were shown to be pairs of protein-coding genes (AGI code genes) and non-protein-coding RNAs from non-AGI transcriptional units [21,24]; however, it has been reported that > 86% of rice bidirectional transcript pairs included a coding sequence in both strands [19]. (ii) The distribution of antisense transcripts in mammals was found to be non-random across the genome [29]. (iii) Expression levels of sense and antisense transcripts can be either positively or negatively correlated [15,21,30,31]. (iv) In *Arabidopsis*, most of the *cis-*NATs are arranged in convergent orientation. Several genes were found to be involved in two *cis-*NATs as a network: one pair is convergent, another is divergent [20,27].

However, there are still some limitations to comprehensively identifying *cis-*NATs. Firstly, the percentage of *cis-*NATs in different eukaryotic genomes, especially in plants, is estimated mainly by the alignment of full-length cDNAs, ESTs and predicted coding sequences to the genome. The transcriptomes are still not sequenced deeply enough to provide all transcripts, including low copy number and non-coding RNAs (ncRNAs). Secondly, as important evidence to determine the regulation of *cis*-NATs by RNA interference, nat-siRNAs are still far from saturated, despite many efforts in plants (rice and *Arabidopsis*) in recent years [26-28,32-35]. Thirdly, it is also essential to accurately quantify the expression levels of sense and antisense transcripts at a global level.

The advent of second-generation sequencing technology enables deep sequencing of transcripts. The paired-end tag sequencing strategy of strand-specific cDNA sequencing technology (ssRNA-seq) has the potential to globally produce abundant and novel transcripts with clear polarity and to accurately assess gene activity [36,37]. Moreover, each cell type has its unique transcriptome, so a single-cell-level description of gene expression and regulation can be instructive concerning cell populations [38,39]. The latest RNA-seq applied to single cells gives more precise transcriptome quantifications than a PCR-based amplification method [40]. Meanwhile, high-throughput sequencing techniques have made it feasible to obtain all small RNAs species, genome-wide, as it can generate hundreds of millions reads in a single sequencing run.

Here, we took advantage of ssRNA-seq technology to deeply sequence cDNAs with clear transcriptional orientations in the model plant species rice (*Oryza sativa* L.). All mRNAs were derived from seedlings grown under normal (seedling mixture, and only epidermal cells as well) and abiotic stressed conditions for assessing rice *cis-*NATs at the best possible resolution. We also deeply sequenced small RNAs to investigate nat-siRNAs from rice seedlings under normal and several stresses conditions. In addition, we tried to sequence transcriptome of rice leaf epidermal cells and evaluate the expression of *cis-*NATs identified in this research. We identified 2292 rice *cis-*NATs with both evidence of gene expression and nat-siRNAs from their overlapping regions. About 54.0% of them were shown to be simultaneously expressed in epidermal cells. Some *cis-*NATs gave rise to nat-siRNAs exclusively in the overlap regions, and some *cis-*NATs seemed to be expressed under specific abiotic stress conditions. This study was the first attempt of applying ssRNA-seq to deeply investigate novel transcripts and revealed widespread occurrence of *cis-*NATs in rice.

## Results

### ssRNA-seq and assembly of rice transcripts

In order to comprehensively identify rice *cis-*NATs, we applied ssRNA-seq to deeply sequence rice cDNAs for assessing transcripts with clear transcriptional orientations. Rice seedlings grown under normal and three abiotic stress conditions (salt, cold and drought treatments) were collected for preparations of mRNA and small RNAs (see Methods), and four strand-specific cDNA libraries were then constructed according to the empirical protocol [37]. In general, a modified RNA-seq method is used for incorporation of deoxy-UTP during second-strand cDNA synthesis and subsequent destruction of the uridine-containing strand in the sequencing library. This enables the identification of transcript orientation. The high-throughput sequencing of strand-specific cDNAs was performed on the Illumina GAIIX. In total, 14.7, 11.5, 14.2 and 13.2 million paired-end reads of 2 × 120 bp with high-quality, which were generated from the untreated and salt, cold and drought stress treatments libraries, respectively, matched unambiguously and uniquely to the rice reference genome [41] (Additional file 1). We estimated the accuracy of transcriptional orientation by comparing the sequencing reads with the annotated gene datasets [42]. About 89.4-95.5% of the mapped reads appeared to be aligned with correct transcriptional orientation, providing strong evidence for ssRNA-seq data in a strand-specific manner. In contrast to ssRNA-seq data, only half of the mapped reads from the previous RNA-seq data were consistent with the gene models at the same transcriptional orientations. In addition, we also deeply sequenced epidermal cells of normal rice seedlings, and about 10.4 million paired-end reads of 2 × 100 bp were generated as high-quality data.

Using the software TopHat and Cufflinks [43-46], we assembled ssRNA-seq data combined with the rice genome annotation [42] into 76,013 transcripts corresponding to 45,844 unique gene loci (including 4873 novel gene loci). Of them, 25,924 were identified as novel transcripts, which were composed of 5063 ncRNA, 16,494 CDS with protein hits and 4367 CDS without any protein hits (Additional file 2).

### Identification of putative cis-NATs in rice

Based on renewed assembled transcripts, we identified putative *cis-*NATs which overlapped, but were opposite, from the same or adjacent gene locus. In total, 5813 pairs of rice *cis-*NATs (Additional file 3) were preliminarily screened out. After excluding those in which either of the pairs encoded a transposon, rRNA, tRNA, snRNA, snoRNA or miRNA, we obtained 3819 putative *cis-*NATs with mean overlapped length of 785 nt (Table 1). According to the directions of the involved transcripts, 2149 (56.3%) *cis-*NATs were categorized in enclosed, 898 (23.5%) in convergent (3^′^-3^′^ overlap), and 772 (20.2%) in divergent (5^′^-5^′^ overlap) orientations. Of rice *cis*-NATs, 36.1% (1378 of 3819) were pairs of protein-coding genes and non-protein-coding RNAs (Table 1). Another 33.4% (1275 of 3819) were a PFAM domain-containing transcript partner of a predicted CDS without any PFAM domain. In general, the majority of *cis-*NATs (3358 or 87.9%) were one-to-one type, i.e. one transcript in a *cis-*NAT pair had only one antisense partner. The remaining 461 *cis-*NATs (composed of 685 transcripts) were involved in networks of 223 *cis*-NAT groups.

**Table 1:** **Statistics of 3819 *cis-*NATs identified in rice**

**Chr.**	**Transcripts**	***cis*-NATs**^**a**^	**Enclosed**^**b**^	**3**^**′**^**-3**^**′**^^**c**^	**5**^**′**^**-5**^**′**^^**d**^	**CDS-p**^**e**^**vs. ncRNA**	**CDS-p vs. CDS-n**^**f**^	**CDS-p vs. CDS-p**	**CDS-n vs. ncRNA**	**CDS-n vs. CDS-n**	**ncRNA vs. ncRNA**
1	10,536	558	301	146	11	205	176	92	39	30	16
2	8,527	440	239	115	86	159	141	63	36	30	11
3	9,197	491	296	120	75	175	156	90	29	22	19
4	6,762	361	185	95	81	100	132	55	27	27	20
5	5,940	290	161	71	58	107	108	34	21	11	9
6	6166	299	170	69	60	111	100	38	23	13	14
7	5,862	278	163	63	52	90	96	30	24	19	19
8	5,290	239	138	51	50	103	74	27	19	10	6
9	4,256	189	100	34	55	70	71	10	16	10	12
10	4,144	221	128	41	52	85	74	24	19	10	9
11	4,861	231	139	47	45	92	75	25	23	7	9
12	4,472	222	129	46	47	81	72	24	20	15	10
Total	76,013	3,819	2,149	898	772	1,378	1,275	512	296	204	154

^a^*cis*-NAT pairs without transposons.

^b^ One transcript being entirely reverse-complementarily overlapped by the other.

^c^ Convergent *cis*-NAT (with 3^′^-ends overlapped).

^d^ Divergent *cis*-NAT (with 5^′^-ends overlapped).

^e^ Coding sequence with PFAM domain-containing.

^f^ Predicted as coding sequence but without any PFAM domain hits.

### Small RNAs and nat-siRNAs in rice

To investigate the complexity of small RNAs in rice, we generated four small RNA libraries from rice seedlings under normal conditions and three abiotic stress treatments (salt, cold and drought), and deeply sequenced the libraries on the Illumina GAIIX. The small RNA libraries were made with an RNA ligation method which produces strand-specific libraries. After removing low-quality reads and those mapped to rRNA, tRNA, sn/snoRNA, mitochondria and chloroplasts, 4,843,040, 3,973,627, 2,894,255 and 5,492,145 distinct small RNAs (representing 17,632,759, 12,923,509, 8,720,251 and 20,069,157 of 48,683,191, 49,254,272, 25,705,840 and 50,192,805 raw reads, respectively) from corresponding untreated, salt, cold and drought conditions, were identified (Additional file 1). Small RNAs of 24-nt were the predominant size class (Additional file 4A). We found that the majority were located in transposon-related regions, followed by upstream, intergenic, downstream, and intron regions (Additional file 4C).

To determine the amount of nat-siRNAs, we searched in the four small RNA libraries that matched uniquely and perfectly to the overlapping regions of the 3819 *cis-*NATs. A total of 180,239 reads corresponding to 90,977 unique small RNAs (normal: 25,420; salt: 18,598; cold: 18,152 and drought: 28,807) were derived from overlapped regions (Additional files 1 and 5). Here, nat-siRNAs showed wide ranges in size of 18-34 nt, with 21-25 nt the most common size (Additional file 4B). The 5^′^-first nucleotide of nat-siRNAs was predominantly adenosine (Additional file 4F), which differed from that of the total small RNAs (Additional file 4E).

### cis-NATs and nat-siRNAs with gene expression evidence under normal or abiotic stress conditions

To gain further insight into regulation of *cis-*NATs, we examined the expression levels of 3358 one-to-one type *cis-*NATs. The 2292 *cis-*NAT pairs were detected as expressed [i.e. Fragments Per Kilobase of exon Models (FPKM) > 0] with both sense and antisense transcripts under normal (1789 pairs), cold (1668), salt (1572) or drought (1668) conditions, and also with nat-siRNAs in an overlapping region (Table 2 and Additional file 6). We found that 166 pairs produced siRNAs exclusively and had more than five unique small RNA reads in the overlapping regions (Additional file 7). To explore whether small RNAs were more enriched in the overlapping compared to non-overlapping regions, we calculated small RNA densities of *cis-*NATs in these two regions. The siRNA densities of 13 *cis-*NATs were > 5 times that in the non-overlapping regions (Additional file 8). We further investigated *cis-*NATs which generated nat-siRNAs with strand bias: 25-28% of *cis-*NAT pairs exhibited strong strand bias in spawning nat-siRNAs with > 5-fold change in both normal and abiotic stresses (Additional file 9A). More than 75% of *cis-*NATs generated small RNAs from different directions with > 2-fold change (Additional file 9B). Interestingly, *cis-*NAT pairs, which were composed of the protein-coding gene partner of non-protein-coding RNAs, overwhelmingly produced small RNAs from the strand of protein-coding transcripts (Additional file 9).

**Table 2:** **Numbers of *cis-*NATs with expression evidence and nat-siRNAs, under four different conditions and in epidermal cells**

	**Both expressed *cis*cbsgNAT pairs**	**Validated in epidermal cells**	**Pairs with siRNAs only in overlap region**^**f**^	**Pairs with siRNAs enriched in overlap region**^**g**^
sd^a^	1,789	1,043	72	9
ST^b^	1,572	949	58	5
CD^c^	1,668	1,003	66	5
DT^d^	1,668	986	75	5
Co-	1,072 (46.7%)^e^	725 (58.6%)^e^	10 (5.9%)^e^	2 (15.4%)^e^
Total	2,292	1,238	166	13

^a^ 14-d-old-seedling.

^b^ 14-d-old-seedling treated with 200 mM NaCl.

^c^ 14-d-old-seedling grown under cold stress at 4°C for 24 h in darkness.

^d^ 14-d-old-seedling treated with 20% PEG-6000.

^e^ The percentage of co-*cis-*NATs in the corresponding total *cis-*NATs was calculated.

^f^ Small RNAs were only enriched in the overlapping regions with more than five reads.

^g^ The density of siRNAs in overlapping regions was > 5 times that in non-overlapping regions.

**Figure 1:**
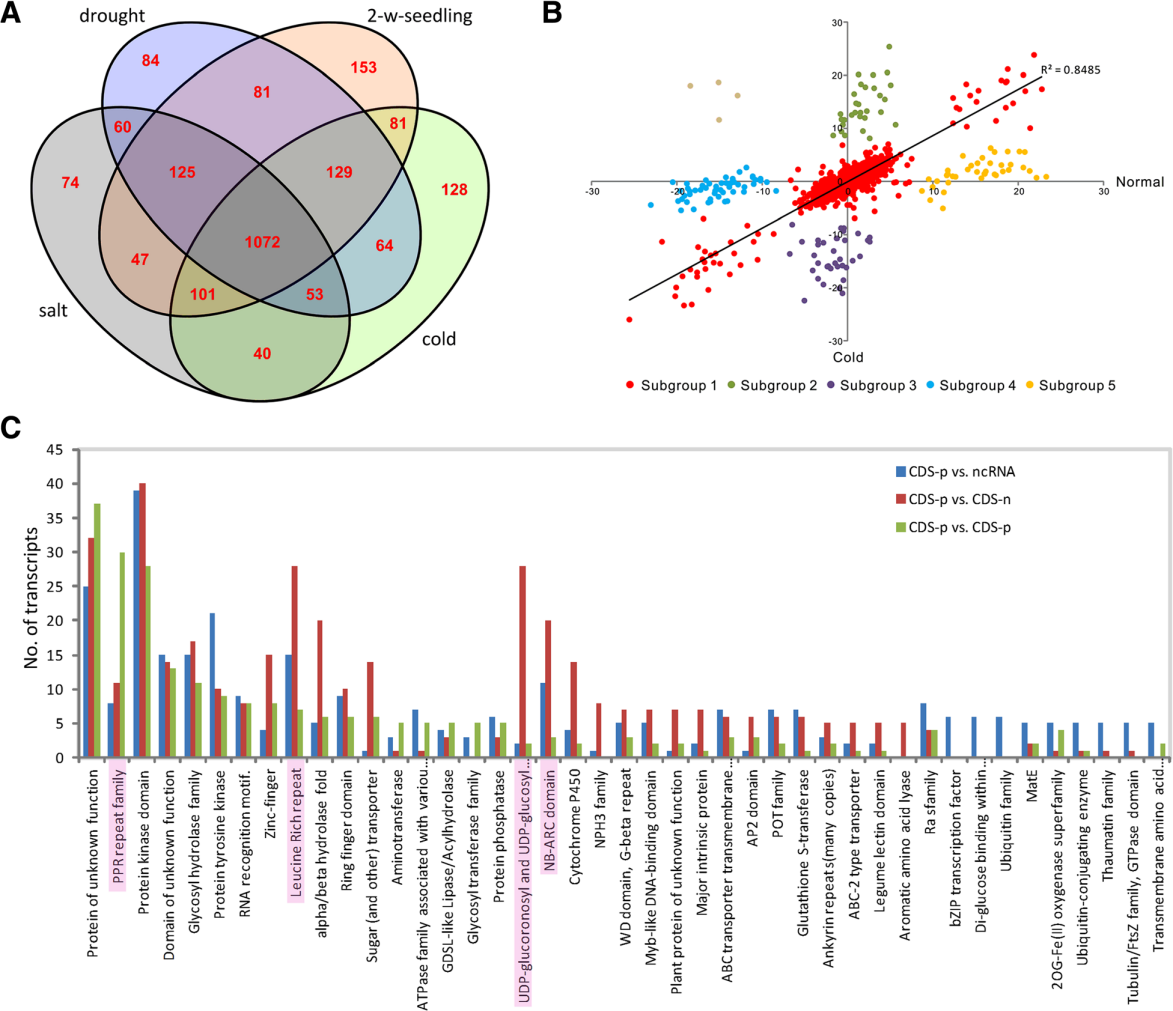
**Overview of 2292 one-to-one *cis-*NATs.** (**A**) Venn diagram showing the shared *cis*-NATs that expressed under normal (control), cold, salt and drought conditions. The numbers of *cis*-NATs that expressed under different conditions are noted in the corresponding parentheses. (**B**) Scatter plot showing comparison of transcripts expression ratio trends of 1072 co-expressed *cis*-NAT pairs between normal and cold stress conditions. Five subgroups 1, 2, 3, 4 and 5 are indicated by red, green, purple, blue and orange points, respectively. (**C**) Three sets of genes are categorized on the basis of components of each *cis*-NAT pair. Set I (blue bar): 767 *cis*-NAT pairs of protein-coding genes and non-protein-coding RNAs; Set II (red bar): 818 *cis*-NAT pairs of protein-coding genes and predicted CDS without any PFAM domain; Set III (green bar): 377 *cis*-NATs composed of both protein-coding genes. Y-axis represents the number of transcripts. X-axis represents 43 protein families that were the major enriched terms in either of three sets. Four proteins of pentatricopeptide repeat (PPR) families, leucine rich repeat, NB-ARC domain and UDP-glucoronosyl and UDP-glucosyl transferase, which exhibited distinctively enriched terms with P < 0.001 in Sets II and III, are highlighted in pink in the X-axis.

Of 2292 *cis-*NATs, 1072 (46.7%) *cis-*NATs were expressed in both normal and abiotic stresses (Figure 1A). The *cis*-NAT pairs could be divided into five subgroups based on a scatter plot comparing transcripts expressional ratio trends of *cis-*NAT pairs between normal and cold stressed conditions (Figure 1B). We also investigated the functional bias among each group of *cis-*NAT pairs through functional domain and expressional profiling analyses. We found that Protein kinase domains were commonly identified in *cis-*NAT pairs among the five subgroups. However, other functional domains and expression levels of sense/antisense transcripts were detected to be associated with different subgroups of *cis*-NAT pairs.

**Figure 2:**
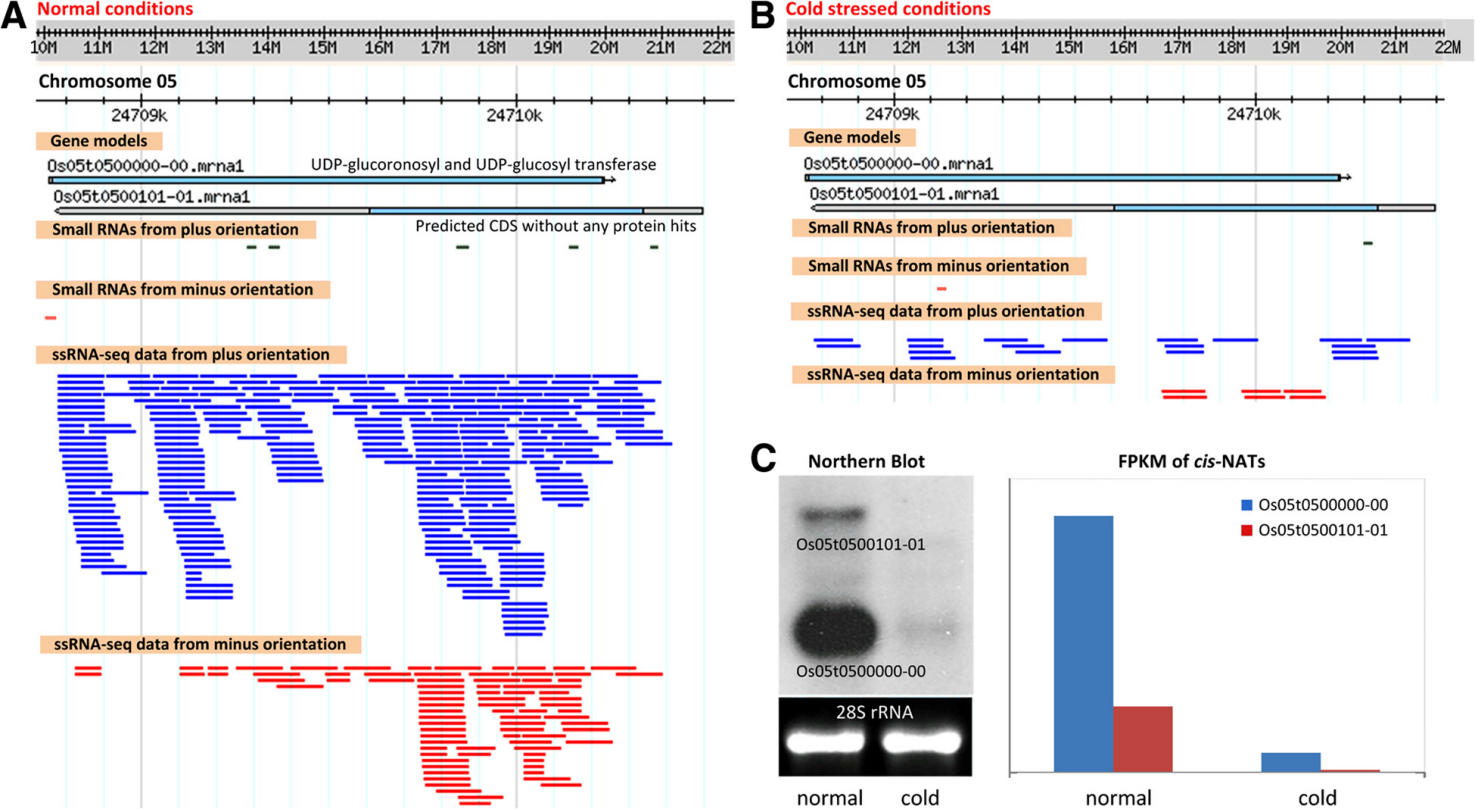
**An example of regulatory small RNAs derived from a *cis*-NAT pair.** (**A**) A *cis-*NAT pair was formed with genes UDP-glucoronosyl and UDP-glucosyl transferase (Os05t0500000-00) and predicted CDS without any protein hits (Os05t0500101-01). Os05t0500000-00 was re-annotated by ssRNA-seq data (short blue lines) from normal condition in the plus strand. Os05t0500101-01 was re-annotated by ssRNA-seq data (short red lines) in the minus strand. The *cis-*NATs spawned five unique small RNAs, mainly from the plus strand (short green lines). (**B**) Under cold-stress conditions, expression of this *cis-*NAT pair was dramatically down-regulated. (**C**) On the left: Northern blot analysis of the *cis-*NATs confirmed differential expression levels under normal and cold treatment. On the right: the figure shows the FPKM results of this *cis-*NAT pair. FPKM refers to Fragments Per Kilobase of exon Models.

Subgroup-1 *cis-*NATs were predominantly associated with the pentatricopeptide repeat (PPR) and Protein tyrosine kinase domains. We also found that the expression levels of the *cis-*NAT pairs of Subgroup-1 under stress conditions were similar to that under normal conditions. Subgroup-2 *cis-*NATs were identified to contain domains of leucine rich repeats and glycosyltransferase. In this subgroup, the expression levels of sense-transcripts of the *cis-*NAT pairs were significantly increased under stress conditions, while expression levels of antisense transcripts were greatly reduced. Thus, expression levels were higher for sense than for antisense transcripts under stress conditions. Subgroup-3 can be classified as the family of alpha/beta hydrolase fold. The expression levels of sense-transcripts of the Subgroup-3 *cis-*NAT pairs were significantly reduced under stress conditions, while expression levels of antisense transcripts significantly increased. Thus, expression levels were lower for sense than for antisense transcripts under stress conditions. Subgroup-4 *cis-*NATs can be classified as the families of the eukaryotic aspartyl protease and sugar (and other) transporter. Subgroup-5 *cis-*NATs were identified to be the families of glycosyl hydrolases and ubiquitin-conjugating enzyme. The remaining four cis-NAT pairs were not classified into any subgroups, as the expression levels of sense transcripts were not correlated with that of anti-sense partner under normal and abiotic conditions. We identified these four *cis-*NAT pairs as abnormal values (Figure 1B; indicated as the brown spots). Subgroup-1 was shown to be represented the major part. Ratio values of 913 *cis-*NAT pairs (Subgroup-1) between normal and cold stressed conditions were highly correlated, with R^2^ > 0.85. Similar results were also observed between normal and salt/drought stressed conditions with R^2^ > 0.93 of 917 pairs and R^2^ > 0.93 of 898 pairs, respectively (Additional file 10). In total, of 1072 *cis-*NAT pairs, 95.5% (1024) showed positive correlations between sense and antisense transcripts. One *cis-*NAT pair (Os05t0500000-00 vs. Os05t0500101-01) was taken as an example to compare expression changes under normal and cold stressed conditions (Figure 2). Northern blot analyses also demonstrated their differential expression levels under abiotic stresses (Figure 2C). Sense transcript Os05t0500000-00 is annotated as an UDP-glucoronosyl and an UDP-glucosyl transferase, which is an important enzyme for catalyzing transportation of sugars. Antisense transcript Os05t0500101-01 had no hits to any protein families.

**Figure 3:**
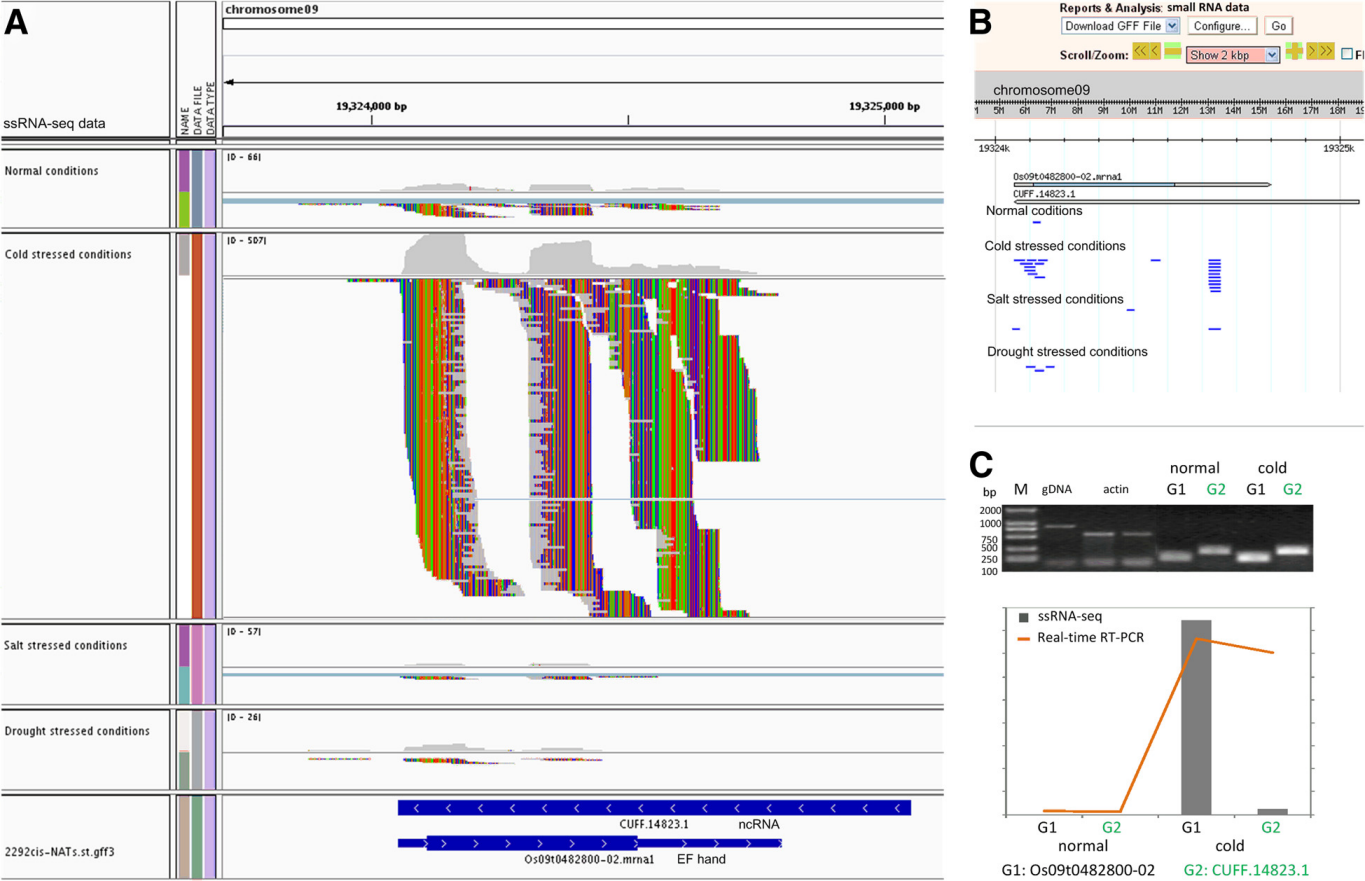
**An example of an overwhelmingly up-regulated *cis-*NAT under abiotic stresses.** (**A**) Expressions of a *cis-*NAT pair of an annotated gene Os09t0482800-02 and a novel ncRNA CUFF.14823.1 are dramatically up-regulated under cold-stress conditions. Expression levels of this *cis-*NAT pair under normal, cold, salt and drought conditions are relatively low and displayed. (**B**) The unique small RNAs that are generated mainly from the plus strand (short blue lines) of the *cis-*NAT pair of the gene Os09t0482800-02 and the ncRNA CUFF.14823.1 are shown. (**C**) Expressions of this *cis*-NAT pair were detected through semi-quantitative RT-PCR analysis. Rice actin expression was used as a control. The PCR products of the *cis-*NAT were amplified by 36 cycles, while actin products were amplified by 26 cycles. Real-time RT-PCR analysis of the *cis-*NATs was used to confirm the differential expressed genes under normal and cold conditions. Expression data from ssRNA-seq (FPKM, Fragments Per Kilobase of exon Models) are represented as gray blocks; and the data from real-time RT-PCR are indicated as orange lines.

**Figure 4:**
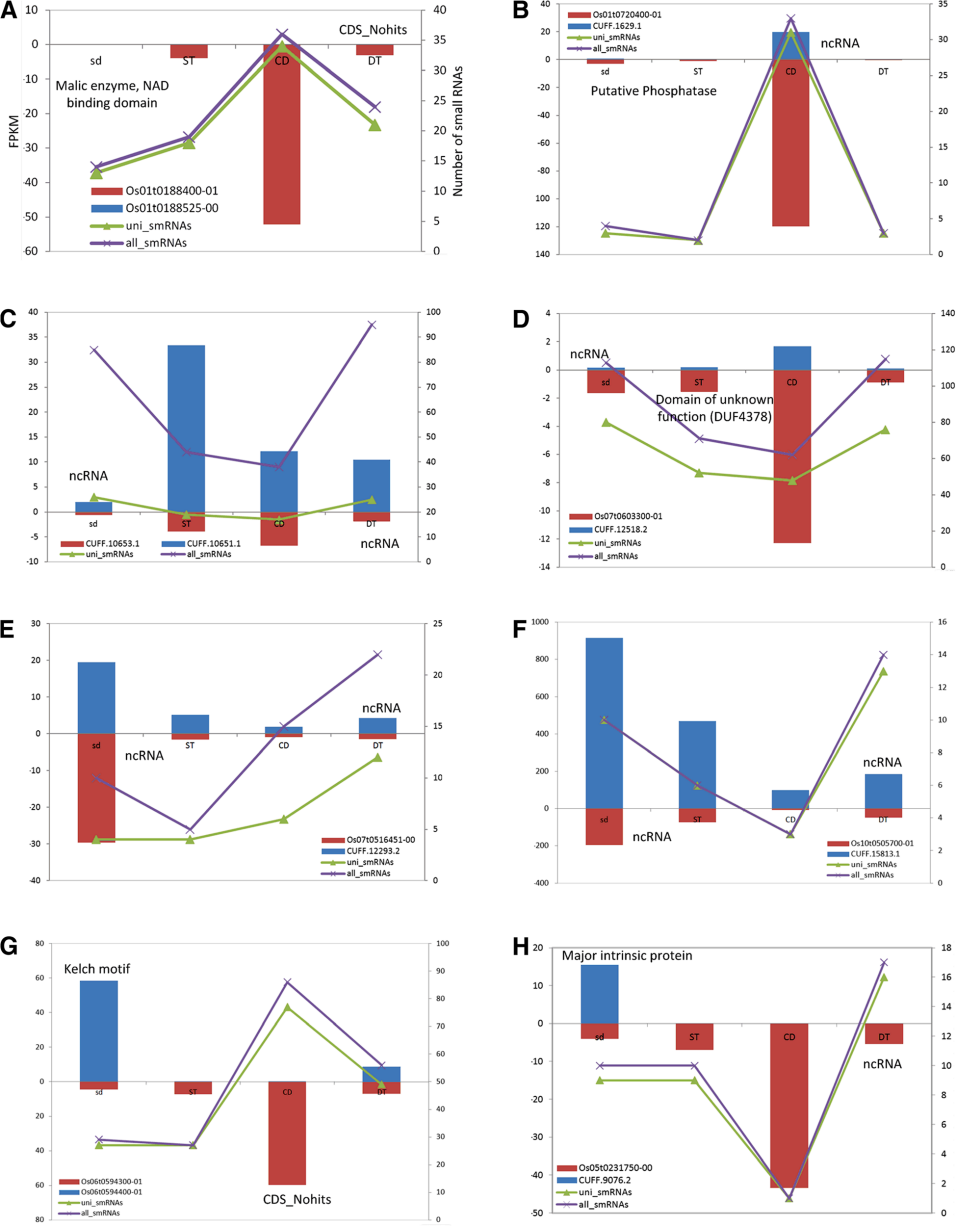
**Expression patterns of eight one-to-one *cis-*NATs and the distribution of small RNAs located in overlapped regions of these gene pairs.** (**A-H**) Expression patterns of eight *cis-*NATs are displayed. The left Y-axis of each graph represents FPKM of each transcript. Blue bars indicate FPKM of plus- transcripts (above the X-axis); red bars indicate FPKM of its partner of minus- transcripts (below the X-axis). The right Y-axis indicates the number of small RNAs located in overlapped regions. The green curves represent unique nat-siRNAs, and the purple curves represent all nat-siRNAs. FPKM refers to Fragments Per Kilobase of exon Models.

The numbers of *cis*-NATs expressed under different conditions were quite different (Figure 1A). The shared *cis*-NATs expressed under normal (control), cold, salt and drought conditions were also identified. Among them, 503 (21.9%) *cis*-NATs were detected preferentially under abiotic stresses compared to normal conditions. There were 84, 74 and 128 *cis*-NAT pairs clearly expressed under drought, salt and cold stresses, respectively. As an example, under cold treatment, sense and antisense transcripts of *cis-*NAT pair Os09t0482800-02 vs. CUFF.14823.1 were both much more expressed than under normal or other stressed conditions (Figure 3). Semi-quantitative RT-PCR and reverse transcription PCR (RT-PCR) were used to validate the expression of the *cis*-NAT pair under normal and cold conditions (Figure 3B). Functional annotation of this *cis-*NAT pair was composed of EF-hand protein (Os09t0482800-02) and ncRNA (CUFF.14823.1). Moreover, 1238 of 2292 (54.0%) *cis-*NATs were expressed in leaf epidermal cells. Of 1238 *cis-*NATs, 725 belonged to co-expressed pairs (Table 2). We further performed analysis of differentially expressed genes (DEGs). In total, we identified 112 *cis-*NATs with DEGs of at least either of each pair, which belonged to different subgroups (Additional file 11). Of them, expression levels of 69 *cis-*NATs (either sense or antisense transcripts) were up-regulated under abiotic stresses, while 46 *cis-*NATs were down-regulated under abiotic stresses. Moreover, the results showed no clear expression pattern between transcripts and nat-siRNAs (Figure 4). The number of nat-siRNAs notably increased along with higher transcriptional expression level under cold-stress conditions (Figure 4A and B); in contrast, some *cis-*NATs generated less nat-siRNAs, along with higher expression levels (Figure 4C and D). In addition, some *cis-*NATs generated nat-siRNAs with more complex expression patterns (Figure 4E-H).

We used an enrichment analysis of PFAM protein families [47] to functionally characterize three sets that could be annotated from 2292 *cis-*NATs. The three sets were 767 *cis-*NAT pairs of protein-coding genes and non-protein-coding RNAs (Set I), 818 *cis-*NAT pairs of protein-coding genes and predicted CDS without any PFAM domain-containing (Set II), and 377 *cis-*NATs composed of both protein-coding genes (Set III), respectively. Roughly, 43 protein families were the major enriched terms in either of three sets (Figure 1C). Of them, four proteins exhibited distinctively enriched terms with *P*-values < 0.001. PPR families were enriched in Set III, leucine rich repeat and NB-ARC domain were significantly reduced in Set III, and UDP-glucoronosyl and UDP-glucosyl transferase were significantly enriched in Set II (Figure 1C).

### Networks formed by cis-NAT groups

We further investigated networks formed by *cis-*NATs in rice, named ‘many-to-many’ *cis-*NATs, i.e. one transcript in a *cis-*NAT had more than one antisense partner. Networks formed by *cis*-NATs reflect the complexity of their post-transcriptional regulation. It has been reported that in *Arabidopsis*, several genes are involved in two *cis-*NATs, one is convergent and the other divergent [20,27]. In rice, we found 461 *cis-*NATs (composed of 685 transcripts) involved in 223 *cis-*NAT groups (Additional file 12). Of them, 209 groups belonged to one-to-two type, nine belonged to one-to-three type, four belonged to two-to-two groups, and one belonged to one-to-four group, respectively. Five types were identified in one-to-two *cis-*NAT groups (Additional file 12). Except 46 groups which were composed of convergent together with divergent *cis-*NATs, it also included 74 enclosed together with enclosed, 40 enclosed together with divergent, 48 enclosed together with convergent, and one convergent together with convergent. Interestingly, we found only 17.4-34.7% *cis-*NAT groups could be detected with simultaneous expression evidence and nat-siRNAs in the same sample from normal, cold, salt or drought conditions; another 29.2-48.6% *cis-*NAT groups were either expressed (Additional file 13). Similar to one-to-one type *cis-*NATs, protein kinase domain, leucine rich repeats, NB-ARC domain and the PPR repeat family were the major enriched PFAM protein domains.

## Discussion

Increasing amounts of evidence indicate that antisense transcripts are frequently functional and play various biological roles using different transcriptional and post-transcriptional gene regulatory mechanisms [1,2,48-53]. Consequently, we tried to advance rice *cis-*NATs analysis through an integrated approach of ssRNA-seq of rice transcriptome and genome-wide *in silico* transcriptome data analysis.

There have been five previous studies using global investigation of rice *cis*-NATs. An analysis of 32,127 full-length rice cDNA sequences showed 687 bidirectional transcript pairs [19]. Comprehensive expression profiles of rice using MPSS technology identified 11,001 antisense signatures corresponding to 8023 annotated genes with highly specific expression patterns [26]. A comparative analysis of *cis*-NATs in eukaryotes identified 1088 rice *cis*-NATs referring to MSU and KOME gene datasets [17]. Combining pyrophosphate-based high-throughput sequencing of rice small RNA and computational analysis of the MSU rice gene models identified 344 *cis*-NATs formed by protein-coding genes [28]. Recently, 767 pairs of *cis*-NATs were identified using the rice genome annotation data (MSU TGAP 6.1) [35]. In this study, we identified 3819 pairs of rice *cis-*NATs. Of them, 2292 were identified as expressed and gave rise to small RNAs from their overlapping regions. The pairs of *cis*-NATs were confirmed to be formed in laser microdissection-captured rice seedling leaf epidermal cells, which were developed from a single-cell tissue. This indicated that antisense transcripts were more widespread and of more complex occurrence than previously found in the rice genome.

Moreover, qRT-PCR and Northern blot analyses confirmed the presence of *cis*-NATs and differential expression levels under normal and abiotic stresses. Nearly 9.7% of rice transcripts were involved in one-to-one or many-to-many *cis-*NATs formation. An overwhelming majority of *cis-*NATs (3358 of 3819, i.e. 87.9%) were of one-to-one type. Small RNAs generated from *cis-*NATs were broadly detected under either normal developmental conditions or stresses. Similar to the situation for mammals [15], we found that the most prominent form of antisense transcription in rice was a non-coding RNA partner of a protein-coding transcript.

We identified 4873 novel gene loci in this study. Compared with previous research, more novel transcribed activity regions with reliable transcriptional orientation were identified by ssRNA-seq. This also indicated that exploration of the rice transcriptome atlas is far from finished. Along with further progress of high-throughput sequencing technology and development of more efficient software for *de novo* transcriptome assembly, more accurate transcriptional units (TUs) could be defined in the future.

It should be pointed out that in our research about 4.5-10.6% of reads appeared to be aligned to antisense transcripts in error (Additional file 1). This is higher than the 3.88% rate reported by Wang et al. [54], who used a slightly modified version of this dUTP method by increasing the incubation time with UDG to enforce the complete degradation of dUTPs.

In consideration of the limited amount of RNAi-related small RNAs (mainly including microRNAs and small interfering RNAs) in public databases, we proposed the following: (i) more microRNAs and siRNAs need to be identified and (ii) more classes of small RNAs that are engaged in RNAi-related machinery or those that are not will be found in future.

## Conclusions

By applying a strand-specific RNA-seq approach, we systematically identified rice *cis-*natural antisense transcripts and putative nat-siRNAs. Our study profiled the most abundant of transcriptional active regions and revealed widespread occurrence of *cis-*NATs in rice, suggesting that regulation through *cis-*NATs and nat-siRNAs could be a common biological phenomenon. Both ssRNA-seq data and small RNA data obtained by high-throughput sequencing technology would supply important resources for further rice transcriptome analysis. It also indicated the feasibility of global investigations of *cis-*NATs by ssRNA-seq in eukaryotic genomes.

## Methods

### Materials and growth conditions

Rice seeds (*O. sativa* ssp. *japonica* cv*.* Nipponbare) were used in all experiments. Seeds germinated at 28°C in darkness for 2 d were transferred to a plant growth chamber to grow for 14 d under controlled conditions (12/12 h and 30/24°C of light/dark cycles) to produce seedlings and epidermal cells. For drought and salt stress treatments, 14-d-old seedlings were incubated in solutions containing 20% PEG-6000 and 200 mM NaCl, for 4 h at 30°C, respectively. For cold treatment, seedlings at the same developmental stage were treated at 4°C for 24 h in darkness.

### Strand-specific cDNA library construction and sequencing

We prepared the strand-specific cDNA libraries according to a protocol [37]. The ssRNA-seq is a simple modification of the RNA-Seq method that incorporates deoxy-UTP during second-strand cDNA synthesis and subsequent destruction of the uridine-containing strand in the sequencing library. Thus it enables identifying the orientation of transcripts. Total RNA was isolated using the TRIzol reagent (Invitrogen), then total genomic DNA was removed from tissues using DNase (New England Biolabs), which was examined by gel electrophoresis. The OligoTex mRNA midi kit (Qiagen) was used to purify poly(A) mRNA from the total RNA samples. Next, mRNAs were fragmented using the RNA fragmentation kit (Ambion). The first cDNA strand was synthesized using random hexamer primers and second-strand cDNA was synthesized where dUTP was used instead of dTTP. In this step, Actinomycin D was used to increase strand specificity by inhibiting second-strand cDNA synthesis. At 15°C 0.5 μl of actinomycin D solution (120 ng/μl), 0.5 μl of RNase OUT (40 units/μl, Invitrogen) and 0.5 μl of SuperScript III polymerase (200 units/μl,Invitrogen) were added to the reaction. Then EB (20 μl) (10mM Tris–Cl, pH 8.5, Qiagen) was added to the reaction, and the dNTPs were removed by purification of the first strand mixture on a self-made 200 μl G-50 gel filtration spin-column equilibrated with 1mM Tris–Cl, pH 7.0. After second strand synthesis and DNA fragmentation process, the sequencing libraries were further constructed by following the manufacturer’s instructions (Illumina). Fragments of 300-400 bp were recovered and purified, and then enriched by PCR for 15 cycles. Each library was loaded into one lane of the Illumina GA IIX for 2 × 120 bp pair-end sequencing at a concentration of 2 pM, except that library of normal seedlings was loaded into two lanes. Image analysis and base calling were finished using the Illumina GA processing pipeline v1.4.

### Laser microdissection (LM)-captured rice seedling leaf epidermal cells and aRNA preparation

Leaves of 15-d-old seedlings of rice variety TP309 grown in a growth chamber at 12/12h and 25/22°C of day/night cycle were used. Seedling leaves were cut into pieces about 5 mm long and immediately processed by microwave-accelerated acetone fixation (BP-111-RS laboratory microwave, Microwave Research & Applications Inc.) and paraffin-embedded as described by Tang et al. [55]. Cross-sections of 10 μm thickness, parallel to leaf vascular bundles, were obtained using a Leica RM2235 rotary microtome. Paraffin-tape transfer system (Instrumedics) and Veritas Microdissection Instrument (Acturus Bioscience) were used for LM-capturing epidermal cells.

We carried out microdissection of epidermal cells, which are a group of cells (a homogenous population cells). There were about 200-500 cells per sample. The total RNAs of epidermal cells were extracted by PicoPure RNA isolation kit (Acturus, CA, USA) with the DNase (RNase-free, Qiagen) treatment. The integrity of the total RNAs was evaluated by Agilent 2100 Bioanalyzer using RNA-6000 Pico LabChips (Agilent Technologies). Because the total RNA quantity of LM-captured epidermal cells was about 10 ng, we used a TargetAmp two-round aminoallyl-aRNA (antisense RNA) amplification kit (Epicentre Biotechnologies, Madison, WI, U.S.A.) with Super-Script III and SuperScript II reverse transcriptases (Invitrogen, Carlsbad, CA U.S.A.) to amplify laser-microdissected RNA [56]. For each amplification, approximately 0.5 ng of total RNA (in a 2-μl volume) was used as starting material and, typically, 5 to 10 μg aminoallyl cRNA was recovered. For evaluating the fidelity of two-round RNA amplification, the TargetAmp one-round aminoallyl-aRNA amplification kit (Epicentre) was used as a one-round amplification control. For each one-round amplification, 400 ng of total RNA was used as starting material and, typically, more than 10 μg of aminoallyl cRNA was recovered. The quality of amplified RNA was also evaluated by Agilent 2100 Bioanalyzer, and only those with a ‘bell-shaped’ curve with peak size > 300 nucleotides were used for RNA-seq.

### Small RNA sequencing

The same materials described above were used for small RNA library construction using the Illumina small RNA preparation kit (v1.5) following the manufacturer’s instructions. Small RNAs of 18-34 bp were enriched by polyacrylamide gel electrophoresis, and ligated to 5^′^ and 3^′^ adapters. The ligation product was reverse-transcribed into cDNA, which was then amplified by 15 PCR cycles and subjected to Illumina’s Solexa proprietary sequencing. Here, each library was loaded into two lanes of the Illumina GA IIX for 35-bp sequencing, except that the library of cold treatment was loaded into one lane.

### Northern analysis and qRT-PCR

The PCR primers and Northern probes were designed for *cis-*NAT validation (Additional file 14). Northern blot analysis was carried out as described [57]. To ensure that each pair of primers had specificity of its transcript, one was selected from the overlapping region of *cis*-NATs, and the other from the flanking region. Total RNA was extracted using the Trizol reagent (Invitrogen) according to the manufacturer’s instructions. After treated with DNaseI (NEB), 5 μg of total RNA was used to synthesize the oligo (dT) primed first-strand cDNA using SuperScript™ II reverse transcriptase (Invitrogen). For semi-quantitative RT-PCR, rice actin expression was used as a control, *cis-*NAT PCR products were amplified by 36 cycles, while actin products were amplified by 26 cycles. Real-time RT-PCR was performed on the Applied Biosystems 7500 real-time PCR System. Diluted cDNA was amplified using SYBR Premix Ex Taq™ (TaKaRa). The expression levels of transcripts were normalized by endogenous eEF-1α (AK061464) transcripts. Three technical replicates were taken for each set.

### Assembly of rice transcripts

All ssRNA-seq data obtained from normal and cold, salt and drought stress conditions were respectively mapped to the rice reference genome [41] using software TopHat [43]. Tolerances were set to allow at most two mismatches for paired-end reads in each alignment; and reads with multiple alignments were ignored. The corresponding outputs together with RAP-DB genome annotation data [42] were subsequently used for transcript assembly to detect known and unannotated transcripts and isoforms by another software package, Cufflinks [44,46]. Then, we integrated these transcripts together according to another command, Cuffmerge, from the Cufflinks package. Finally, referring to the renewed gene models, we calculated FPKM (Fragments Per Kilobase of exon Models) of each transcript under normal, abiotic stressed conditions, and leaf epidermal cells as well [45]. Open reading frames (ORFs) of novel transcripts were predicted using the ‘getorf’ program of EMBOSS package [58], with the longest ORF extracted for each transcript. For functional annotation, all transcripts were searched against the PFAM database [47] using HMMER v3.0 (E-value < 0.0001) [59]. The MSU Oryza Repeat Database [60] was used to determine transposable element coordinates on the rice pseudomolecules and all transcribed regions, which were annotated using RepeatMasker [61].

### Identification of cis-NATs and nat-siRNAs

The renewed gene annotation results were applied as models. Those assembled genes with uncertain transcriptional orientation were filtered out first. To profile all potential *cis*-NAT pairs in the rice genome, transcripts that originated from the same locus but from opposite strands and with non-redundant overlapped length > 25 nt were chosen. From each group of sense-antisense pairs extracted, we selected as representative pairs those with the longest exonic overlap. We wrote perl scripts to classify *cis-*NAT types: convergent, divergent or enclosed. Furthermore, we examined expression levels of all assembled genes using ssRNA-seq data. Thus, we could identify expressed transcripts from putative *cis-*NATs. According to the number of antisense partners in each *cis-*NAT pair, two types of *cis*-NATs were defined. One was one-to-one type, i.e. one transcript in a *cis*-NAT pair has only one antisense partner. Another is many-to-many *cis*-NATs, i.e. one transcript in a *cis*-NAT has more than one antisense partner.

Raw data of small RNA reads from four corresponding small RNA libraries were aligned to the rice genome [41] by SMALT v0.5.7 with default parameters [62]. Mapping scores below the threshold were not reported. After removing the 5^′^- and 3^′^-adapters, only those continuously and perfectly matched reads with length of 18-34 bp were extracted for further analysis. Reads with multiple alignments or with any mismatches were completely filtered out. Next, we discarded those sequences that could be unambiguously mapped to rRNA, tRNA, sn/snoRNA, mitochondria and chloroplasts. We calculated small RNA densities according to a published method [28]. Small RNAs (at least one unique read) which fully located in overlapped regions of *cis*-NATs were extracted as putative nat-siRNAs. The *cis*-NATs were identified from the set of DEGs across normal and three abiotic-stress libraries. The analysis was carried out based on the following criteria: FPKM of transcripts were used for comparison by computing fold changes (with absolute value ≥ 2) and Fisher’s exact test (p < 0.001) according to an ‘R’ statistical package named ‘DEGseq’ [63].

In addition, R^2^ is the square of the Pearson product-moment correlation coefficient relating the regressor and the response variable. R^2^ gives some information about the goodness-of-fit of a model. In regression, R^2^ is a statistical measure of how well the regression line approximates the real data points. ‘R’ software was used to calculate R^2^[64] of *cis-*NATs between normal and stressed conditions.

### Data release

The raw sequences were deposited in the EBI European Nucleotide Archive with accession number E-MTAB-721 (http://www.ebi.ac.uk/arrayexpress/browse.html?keywords=E-MTAB-721) and ERP001962 (http://www.ebi.ac.uk/ena/data/search?query=ERP001962). The assembled transcripts can be freely downloaded xfrom http://www.ncgr.ac.cn/scientific_data.asp.

## Competing interests

The authors declare that they have no competing interests.

## Authors’ contributions

TL and BH initiated the project; TL and BH designed the experiments; GL, YG, YZ, ZZ, WL, WT and QF carried out the experiments; TL, CZ and YZ performed the computational analyses; TL, YZ and BH participated in the experimental analyses. TL and BH coordinated the research. TL and BH wrote the paper. All authors have read and approved the manuscript for publication.

## Supplementary Material

Additional file 1 Summary of pair-end reads of ssRNA-seq and small RNAs from normal and three abiotic stress conditions. Click here for file

Additional file 2 Statistics of rice transcripts. Click here for file

Additional file 3 **Summary of 5813 putative *cis-*NATs in rice.** This table lists all *cis-*NATs identified from rice renewed assembled transcripts. Each column represents plus transcripts (transcriptional orientations are the same as the reference), its length, minus transcripts, length, overlapped length and detailed overlapped locations in rice genome (IRGSP v5.0) as indicated. Click here for file

Additional file 4 **Overview of small RNAs in rice.** (A) Distribution of the lengths of all unique small RNAs generated by high-throughput sequencing from rice under four different conditions. (B) Length distribution of nat-siRNAs. (C) Distribution of all unique small RNAs located in different sequence components. (D) Distribution of nat-siRNAs located in different sequence components. The blue, red, green and purple bars represent small RNAs or nat-siRNAs from normal, salt stressed, cold stressed and drought stressed conditions, respectively (A-D). (E) First-nucleotide distribution of all unique small RNAs under four different conditions. (F) First-nucleotide distribution of nat-siRNAs under four different conditions. Click here for file

Additional file 5 **Sequences of small RNAs mapped to the overlapped regions of*cis-*NATs.** The 25,420, 18,598, 18,152 and 28,807 unique small RNAs from normal, salt, cold and drought conditions, respectively, are shown to be perfectly mapped to the overlapped regions of *cis-*NATs. Click here for file

Additional file 6 **Details of 2292 expressed*cis-*NATs.** The detailed information of 2292 *cis*-NAT pairs with expression evidence (i.e. FPKM > 0) of both sense and antisense transcripts and with nat-siRNAs in the overlapping region under normal (1789 pairs), cold (1668), salt (1572) and drought (1668) conditions are shown. Columns 1-4 and 9-11 represent *cis*-NATs type, transcripts, protein domain and the length of transcripts, respectively. Columns 5-8 and 12-15 represent FPKM of transcripts under normal, salt, cold and drought conditions, respectively. Column 16 shows the overlapped length. Columns 17-20 show the number of corresponding nat-siRNAs located in the overlapped regions. The rest of the columns are the detailed strand bias of nat-siRNAs from four different conditions. Click here for file

Additional file 7 **Lists of 166*cis-*NATs that produced nat-siRNAs exclusively in the overlapping regions with more than five unique small RNAs from four different conditions.** Of 166 *cis-*NATs, 72, 58, 66 and 75 *cis-*NATs were obtained from normal, salt, cold and drought treatments, respectively. These *cis*-NATs are listed separately in four tables, respectively indicated by blue, orange, red and green colour. Click here for file

Additional file 8 **Lists of 13*cis-*NATs that produced nat-siRNAs more enriched in the overlapping regions than the non-overlapping regions.** Of 13 *cis-*NATs, nine, six, five and five *cis-*NATs obtained from normal, salt, cold and drought treatments, respectively. These *cis*-NATs are listed separately in four tables, respectively indicated by blue, orange, red and green colour. Click here for file

Additional file 9 **Strand bias of*cis-*NATs that gave rise to nat-siRNAs.** The strand bias is computed among different types of *cis-*NAT pairs under four different conditions. A total of nine types are listed. Here, plus indicates that the orientation of transcripts are the same as the reference genome, minus indicates opposite orientation of transcripts. One strand means only one strand of the *cis*-NATs gave rise to small RNAs. The plus/minus or minus/plus means one strand of the *cis*-NATs spawns at least five (part A) or two (part B) fold more small RNAs than the other. Click here for file

Additional file 10 **Scatter plots of expression of 1072*cis-*NATs.** The two scatter plots compare transcripts expression ratio trends of 1072 co-expressed *cis-*NAT pairs between normal and drought-stress conditions (A), and between normal and salt-stress conditions (B). The results show five subgroups: red, green, purple, blue and orange spots represent subgroups 1-5, respectively. Click here for file

Additional file 11 **Lists of 112*cis-*NATs with DEGs.** Differentially Expressed Genes (DEGs) were identified in one-to-one *cis-*NATs under salt, drought and cold treatments by comparing their FPKM with that under normal conditions. The gene names, FPKM, fold change (with absolute value > 2) and p-value (< 0.001) are listed. Click here for file

Additional file 12 **All many-to-many*cis-*NAT groups.** In total, 223 *cis-*NAT groups were identified as many-to-many type. Of them, 209 groups belonged to one-to-two type, nine belonged to one-to-three type, four belonged to two-to-two groups, and one belonged to the one-to-four group. The meaning of each column is as given in Additional file 6. Click here for file

Additional file 13 **Statistics of 209 networks formed by*cis*-NAT groups.** The numbers of one-to-two *cis*-NAT groups with expression evidence under normal, salt, cold and drought conditions are shown. These one-to-two *cis-*NAT groups were divided into five classes according to their *cis-*NATs types. Here, ‘All EXP’ indicates all *cis*-NAT groups with expression evidence (FPKM > 0) of both sense and antisense transcript, and with nat-siRNAs (number of small RNAs > 1) in overlapping region as well. ‘Either EXP’ indicates either of *cis*-NAT groups with expression evidence of both sense and antisense transcript, and with nat-siRNAs in the overlapping region as well. Click here for file

Additional file 14 **Primers designed for real-time RT-PCR and Northern Blots in this research.** (DOCX 13 kb) Click here for file

## References

[B1] Borsani O Zhu J Verslues PE Sunkar R Zhu JK Endogenous siRNAs derived from a pair of natural cis-antisense transcripts regulate salt tolerance in Arabidopsis Cell 2005 123 1279 1291 10.1016/j.cell.2005.11.035 16377568 PMC3137516

[B2] Ron M Alandete SM Eshed WL Fletcher JC McCormick S Proper regulation of a sperm-specific cis-nat-siRNA is essential for double fertilization in Arabidopsis Genes Dev 2011 24 1010 1021 20478994 10.1101/gad.1882810 PMC2867206

[B3] Faghihi MA Wahlestedt C Regulatory roles of natural antisense transcripts Nat Rev Mol Cell Biol 2009 10 637 643 10.1038/nrm2738 19638999 PMC2850559

[B4] Prescott EM Proudfoot NJ Transcriptional collision between convergent genes in budding yeast Proc Natl Acad Sci USA 2002 99 8796 8801 10.1073/pnas.132270899 12077310 PMC124378

[B5] Aravin AA Naumova NM Tulin AV Vagin VV Rozovsky YM Gvozdev VA Double-stranded RNA-mediated silencing of genomic tandem repeats and transposable elements in the D. melanogaster germline Curr Biol 2001 11 1017 1027 10.1016/S0960-9822(01)00299-8 11470406

[B6] Tufarelli C Stanley JA Garrick D Sharpe JA Ayyub H Wood WG Higgs DR Transcription of antisense RNA leading to gene silencing and methylation as a novel cause of human genetic disease Nat Genet 2003 34 157 165 10.1038/ng1157 12730694

[B7] Katiyar-Agarwal S Morgan R Dahlbeck D Borsani O Villegas AJ Zhu JK Staskawicz BJ Jin H A pathogen-inducible endogenous siRNA in plant immunity Proc Natl Acad Sci USA 2006 103 18002 18007 10.1073/pnas.0608258103 17071740 PMC1693862

[B8] Hastings ML Milcarek C Martincic K Peterson ML Munroe SH Expression of the thyroid hormone receptor gene, erbAalpha, in B lymphocytes: alternative mRNA processing is independent of differentiation but correlates with antisense RNA levels Nucleic Acids Res 1997 25 4296 4300 10.1093/nar/25.21.4296 9336460 PMC147039

[B9] Peters NT Rohrbach JA Zalewski BA Byrkett CM Vaughn JC RNA editing and regulation of Drosophila 4f-rnp expression by sas-10 antisense read through mRNA transcripts RNA 2003 9 698 710 10.1261/rna.2120703 12756328 PMC1370437

[B10] Lehner B Williams G Campbell RD Sanderson CM Antisense transcripts in the human genome Trends Genet 2002 18 63 65 10.1016/S0168-9525(02)02598-2 11818131

[B11] Shendure J Church GM Computational discovery of sense-antisense transcription in the human and mouse genomes Genome Biol 2002 3 research0044.1 research0044.14 10.1186/gb-2002-3-9-research0044 12225583 PMC126869

[B12] Chen J Sun M Kent WJ Huang X Xie H Wang W Zhou G Shi RZ Rowley JD Over 20% of human transcripts might form sense-antisense pairs Nucleic Acids Res 2004 32 4812 4820 10.1093/nar/gkh818 15356298 PMC519112

[B13] Kiyosawa H Yamanaka I Osato N Kondo S Hayashizaki Y RIKEN GER Group, GSL Members Antisense transcripts with FANTOM2 clone set and their implication for gene regulation Genome Res 2003 13 1324 1334 10.1101/gr.982903 12819130 PMC403655

[B14] Yelin R Dahary D Sorek R Levanon EY Goldstein O Shoshan A Diber A Biton S Tamir Y Khosravi R Nemzer S Pinner E Walach S Bernstein J Savitsky K Rotman G Widespread occurrence of antisense transcription in the human genome Nat Biotechnol 2003 21 379 386 10.1038/nbt808 12640466

[B15] RIKEN Genome Exploration Research Group and Genome Science Group (Genome Network Project Core Group). FANTOM Consortium Antisense transcription in the mammalian transcriptome Science 2005 309 1564 1566 16141073 10.1126/science.1112009

[B16] Zhang Y Liu XS Liu QR Wei L Genome-wide in silico identification and analysis of cis natural antisense transcripts (cis-NATs) in ten species Nucleic Acids Res 2006 34 3465 3475 10.1093/nar/gkl473 16849434 PMC1524920

[B17] Numata K Okada Y Saito R Kiyosawa H Kanai A Tomita M Comparative analysis of cis-encoded antisense RNAs in eukaryotes Gene 2007 392 134 141 10.1016/j.gene.2006.12.005 17250976

[B18] Yamada K Lim J Dale JM Chen H Shinn P Palm CJ Southwick AM Wu HC Kim C Nguyen M Pham P Cheuk R Karlin-Newmann G Liu SX Lam B Sakano H Wu T Yu G Miranda M Quach HL Tripp M Chang CH Lee JM Toriumi M Chan MM Tang CC Onodera CS Deng JM Akiyama K Ansari Y Empirical analysis of transcriptional activity in the Arabidopsis genome Science 2003 302 842 846 10.1126/science.1088305 14593172

[B19] Osato N Yamada H Satoh K Ooka H Yamamoto M Suzuki K Kawai J Carninci P Ohtomo Y Murakami K Matsubara K Kikuchi S Hayashizaki Y Antisense transcripts with rice full-length cDNAs Genome Biol 2003 5 R5 10.1186/gb-2003-5-1-r5 14709177 PMC395737

[B20] Wang XJ Gaasterland T Chua NH Genome-wide prediction and identification of cis-natural antisense transcripts in Arabidopsis thaliana Genome Biol 2005 6 R30 10.1186/gb-2005-6-4-r30 15833117 PMC1088958

[B21] Matsui A Ishida J Morosawa T Mochizuki Y Kaminuma E Endo TA Okamoto M Nambara E Nakajima M Kawashima M Satou M Kim JM Kobayashi N Toyoda T Shinozaki K Seki M Arabidopsis transcriptome analysis under drought, cold, high-salinity and ABA treatment conditions using a tiling array Plant Cell Physiol 2008 49 1135 1149 10.1093/pcp/pcn101 18625610

[B22] Coram TE Settles ML Chen X Large-scale analysis of antisense transcription in wheat using the Affymetrix GeneChip Wheat Genome Array BMC Genomics 2009 10 253 10.1186/1471-2164-10-253 19480707 PMC2694213

[B23] Grigoriadis A Oliver GR Tanney A Kendrick H Smalley MJ Jat P Neville AM Identification of differentially expressed sense and antisense transcript pairs in breast epithelial tissues BMC Genomics 2009 10 324 10.1186/1471-2164-10-324 19615061 PMC2721853

[B24] Okamoto M Tatematsu K Matsui A Morosawa T Ishida J Tanaka M Endo TA Mochizuki Y Toyoda T Kamiya Y Shinozaki K Nambara E Seki M Genome-wide analysis of endogenous abscisic acid-mediated transcription in dry and imbibed seeds of Arabidopsis using tiling arrays Plant J 2010 62 39 51 10.1111/j.1365-313X.2010.04135.x 20088898

[B25] Meyer BC Vu TH Tej SS Ghazal H Matvienko M Agrawal V Ning J Haudenschild CD Analysis of the transcriptional complexity of Arabidopsis thaliana by massively parallel signature sequencing Nat Biotechnol 2004 22 1006 1011 10.1038/nbt992 15247925

[B26] Nobuta K Venu RC Lu C Beló A Vemaraju K Kulkarni K Wang W Pillay M Green PJ Wang GL Meyers BC An expression atlas of rice mRNAs and small RNAs Nat Biotechnol 2007 25 473 477 10.1038/nbt1291 17351617

[B27] Jin H Vacic V Girke T Lonardi S Zhu JK Small RNAs and the regulation of cis-natural antisense transcripts in Arabidopsis BMC Mol Biol 2008 9 6 10.1186/1471-2199-9-6 18194570 PMC2262095

[B28] Zhou X Sunkar R Jin H Zhu JK Zhang W Genome-wide identification and analysis of small RNAs originated from natural antisense transcripts in Oryza sativa Genome Res 2009 19 70 78 18971307 10.1101/gr.084806.108 PMC2612963

[B29] He Y Vogelstein B Velculescu VE Papadopoulos N Kinzler KW The antisense transcriptomes of human cells Science 2008 322 1855 1857 10.1126/science.1163853 19056939 PMC2824178

[B30] Henz SR Cumbie JS Kasschau KD Lohmann JU Carrington JC Weigel D Schmid M Distinct expression patterns of natural antisense transcripts in Arabidopsis Plant Physiol 2007 144 1247 1255 10.1104/pp.107.100396 17496106 PMC1914114

[B31] Okamoto M Seki M Expression profile and 5′-terminal structure of Arabidopsis antisense transcripts expressed in seed Plant Signal Behav 2011 6 691 693 10.4161/psb.6.5.14976 21448002 PMC3172838

[B32] Xie Z Johansen LK Gustafson AM Kasschau KD Lellis AD Zilberman D Jacobsen SE Carrington JC Genetic and functional diversification of small RNA pathways in plants PLoS Biol 2004 2 E104 10.1371/journal.pbio.0020104 15024409 PMC350667

[B33] Rajagopalan R Vaucheret H Trejo J Bartel DP A diverse and evolutionarily fluid set of microRNAs in Arabidopsis thaliana Genes Dev 2006 20 3407 3425 10.1101/gad.1476406 17182867 PMC1698448

[B34] Kasschau KD Fahlgren N Chapman EJ Sullivan CM Cumbie JS Givan SA Carrington JC Genome-wide profiling and analysis of Arabidopsis siRNAs PLoS Biol 2007 5 e57 10.1371/journal.pbio.0050057 17298187 PMC1820830

[B35] Zhang X Xia J Lii YE Barrera-Figueroa BE Zhou X Gao S Lu L Niu D Chen Z Leung C Wong T Zhang H Guo J Li Y Liu R Liang W Zhu JK Zhang W Jin H Genome-wide analysis of plant nat-siRNAs reveals insights into their distribution, biogenesis and function Genome Biol 2012 13 R20 10.1186/gb-2012-13-3-r20 22439910 PMC3439971

[B36] Wang Z Gerstein M Snyder M RNA-Seq: a revolutionary tool for transcriptomics Nat Rev Genet 2009 10 57 63 10.1038/nrg2484 19015660 PMC2949280

[B37] Parkhomchuk D Borodina T Amstislavskiy V Banaru M Hallen L Krobitsch S Lehrach H Soldatov A Transcriptome analysis by strand-specific sequencing of complementary DNA Nucleic Acids Res 2009 37 e123 10.1093/nar/gkp596 19620212 PMC2764448

[B38] Wang D Bodovitz S Single cell analysis: the new frontier in ‘omocs’ Trends Biotechol 2010 28 281 290 10.1016/j.tibtech.2010.03.002 PMC2876223 20434785

[B39] Tang F Lao L Surani A Development and applications of single-cell transcriptom analysis Nat Methods 2011 8 S6 S11 10.1038/nchembio.740 21451510 PMC3408593

[B40] Hashimshony T Wagner F Sher N Yanai I CEL-Seq: Single-Cell RNA-Seq by Multiplexed Linear Amplification Cell Rep 2012 2 1 8 10.1016/j.celrep.2012.05.015 22939981

[B41] IRGSPv5.0 http://rgp.dna.affrc.go.jp/IRGSP/Build5/build5.html

[B42] RAP-DBhttp://rapdblegacy.dna.affrc.go.jp/download/index.html

[B43] Trapnell C Pachter L Salzberg SL TopHat: discovering splice junctions with RNA-Seq Bioinformatics 2009 25 1105 1111 10.1093/bioinformatics/btp120 19289445 PMC2672628

[B44] Trapnell C Williams BA Pertea G Mortazavi A Kwan G van Baren MJ Salzberg SL Wold BJ Pachter L Transcript assembly and quantification by RNA-Seq reveals unannotated transcripts and isoform switching during cell differentiation Nat Biotechnol 2010 28 511 515 10.1038/nbt.1621 20436464 PMC3146043

[B45] Roberts A Trapnell C Donaghey J Rinn JL Pachter L Improving RNA-Seq expression estimates by correcting for fragment bias Genome Biol 2011 12 R22 10.1186/gb-2011-12-3-r22 21410973 PMC3129672

[B46] Roberts A Pimentel H Trapnell C Pachter L Identification of novel transcripts in annotated genomes using RNA-Seq Bioinformatics 2011 27 2325 2329 10.1093/bioinformatics/btr355 21697122

[B47] Bateman A Birney E Cerruti L Durbin R Etwiller L Eddy SR Griffiths-Jones S Howe KL Marshall M Sonnhammer EL The Pfam protein families database Nucleic Acids Res 2004 32 D138 D141 10.1093/nar/gkh121 14681378 PMC308855

[B48] Shearwin KE Callen BP Egan JB Transcriptional interference - a crash course Trends Genet 2005 21 339 345 10.1016/j.tig.2005.04.009 15922833 PMC2941638

[B49] Crampton N Bonass WA Kirkham J Rivetti C Thomson NH Collision events between RNA polymerases in convergent transcription studied by atomic force microscopy Nucleic Acids Res 2006 34 5416 5425 10.1093/nar/gkl668 17012275 PMC1636470

[B50] Yu W Gius D Onyango P Muldoon-Jacobs K Karp J Feinberg AP Cui H Epigenetic silencing of tumour suppressor gene p15 by its antisense RNA Nature 2008 451 202 206 10.1038/nature06468 18185590 PMC2743558

[B51] Kanduri C Functional insights into long antisense noncoding RNA Kcnq1ot1 mediated bidirectional silencing RNA Biol 2008 5 208 211 18971626 10.4161/rna.7113

[B52] Ohhata T Hoki Y Sasaki H Sado T Crucial role of antisense transcription across the Xist promoter in Tsix-mediated Xist chromatin modification Development 2008 135 227 235 18057104 10.1242/dev.008490

[B53] Richards M Tan SP Chan WK Bongso A Reverse serial analysis of gene expression (SAGE) characterization of orphan SAGE tags from human embryonic stem cells identifies the presence of novel transcripts and antisense transcription of key pluripotency genes Stem Cells 2006 24 1162 1173 10.1634/stemcells.2005-0304 16456128

[B54] Wang L Si Y Dedow LK Shao Y Liu P Brutnell TP A low-cost library construction protocol and data analysis pipeline for Illumina-based strand-specific multiplex RNA-seq PLoS One 2011 6 e26426 10.1371/journal.pone.0026426 22039485 PMC3198403

[B55] Tang WS Coughlan S Crane E Beatty M Duvick J The application of laser microdissection to in planta gene expression profiling of the maize anthracnose stalk rot fungus Colletotrichum graminicola Mol Plant Microbe Interact 2006 19 1240 1250 10.1094/MPMI-19-1240 17073306

[B56] Tang X Zhang ZY Zhang WJ Zhao XM Li X Zhang D Liu QQ Tang WH Global Gene Profiling of Laser-Captured Pollen Mother Cells Indicates Molecular Pathways and Gene Subfamilies Involved in Rice Meiosis Plant Physiol 2010 154 1855 1870 10.1104/pp.110.161661 20959420 PMC2996036

[B57] Huang J Takano T Akita S Expression of α-expansin genes in young seedlings of rice (Oryza sativa L.) Planta 2000 211 467 473 10.1007/s004250000311 11030545

[B58] Rice P Longden I Bleasby A EMBOSS: the European molecular biology open software suite Trends Genet 2000 16 276 277 10.1016/S0168-9525(00)02024-2 10827456

[B59] HMMERhttp://hmmer.janelia.org/

[B60] MSU 6.0 repeat database ftp://ftp.plantbiology.msu.edu/pub/data/Eukaryotic_Projects/o_sativa/annotation_dbs/pseudomolecules/version_6.0

[B61] RepeatMaskerhttp://www.repeatmasker.org

[B62] SMALT version 0.5.7 ftp://ftp.sanger.ac.uk/pub/users/hp3/

[B63] Wang L Feng Z Wang X Wang X Zhang X DEGseq: An R package for identifying differentially expressed genes from RNA-seq data Bioinformatics 2010 26 136 138 10.1093/bioinformatics/btp612 19855105

[B64] R software packagehttp://www.r-project.org/

