# Supplementary material for: Strand-specific RNA-seq reveals widespread occurrence of novel cis-natural antisense transcripts in rice
Source: BMC Genomics. 2012 Dec 22;13:721. doi: 10.1186/1471-2164-13-721 (PMC3549290; doi:10.1186/1471-2164-13-721)
Supplement: Additional file 1 — Summary of pair-end reads of ssRNA-seq and small RNAs from normal and three abiotic stress conditions. [file 1471-2164-13-721-S1.docx]

**Additional file 1.** Summary of pair-end reads of ssRNA-seq and small RNAs from normal and three abiotic stressed conditions.

|  | sd^a^ | ST^b^ | CD^c^ | DT^d^ | epidermal cells |
| --- | --- | --- | --- | --- | --- |
| Raw data of ssRNA-seq (No. of PEs ^e^) | 36,576,657 | 31,409,931 | 33,611,228 | 30,960,975 | 95,559,590 |
| Accuracy of sequenced transcripts orientation (%) | 89.4 | 93.1 | 92.0 | 95.5 | 98.7 |
| No. of PEs with unique matches and good quality | 14,675,262 | 11,529,137 | 14,194,065 | 13,167,041 | 10,356,844 |
| Raw data of small RNAs (No. of reads) | 48,683,191 | 49,254,272 | 25,705,840 | 50,192,805 | - |
| No. of 18nt-34nt small RNAs perfectly matched to the reference | 17,632,759 | 12,923,509 | 8,720,251 | 20,069,157 | - |
| No. of 18nt-34nt unique small RNAs | 4,843,040 | 3,973,627 | 2,894,255 | 5,492,145 | - |
| No. of nat-siRNAs perfectly matched to overlapped regions of cis-NATs | 51,594 | 33,813 | 29,470 | 65,362 | - |
| No. of unique nat-siRNAs | 25,420 | 18,598 | 18,152 | 28,807 | - |

^a^rice 14-day-old-seedling.

^b^rice 14-day-old-seedling treated with 200 mM NaCl.

^c^rice 14-day-old-seedling grown under cold stress at 4°C for 24 h in dark.

^d^rice 14-day-old-seedling treated with 20% PEG-6000.
