# Supplementary material for: Strand-specific RNA-seq reveals widespread occurrence of novel cis-natural antisense transcripts in rice
Source: BMC Genomics. 2012 Dec 22;13:721. doi: 10.1186/1471-2164-13-721 (PMC3549290; doi:10.1186/1471-2164-13-721)
Supplement: Additional file 2 — Statistics of rice transcripts. [file 1471-2164-13-721-S2.docx]

**Additional file 2.** Statistics of rice transcripts.

| Definition | Number |
| --- | --- |
| Assembled transcripts | 76,013 |
| *Transcripts from RAP-DB (including predictions)* | 52,045 (40,971 gene loci) |
| *Novel transcripts* | 25,924 |
| Novel gene loci | 4,873 (11,428 transcripts) |
| Putative ncRNAs (no ORFs) | 12,744 |
| *Novel putative ncRNAs* | 5,063 |
| PFAM domain-containing protein (ORFs) | 30,601 |
| *Novel PFAM domain-containing protein* | 16,494 |
| ORFs without any PFAM domain-containing (ORFs) | 11,807 |
| *Novel ORFs without any PFAM domain-containing* | 4,367 |
