# Supplementary figures and images for: Strand-specific RNA-seq reveals widespread occurrence of novel cis-natural antisense transcripts in rice
Source: BMC Genomics. 2012 Dec 22;13:721. doi: 10.1186/1471-2164-13-721 (PMC3549290; doi:10.1186/1471-2164-13-721)

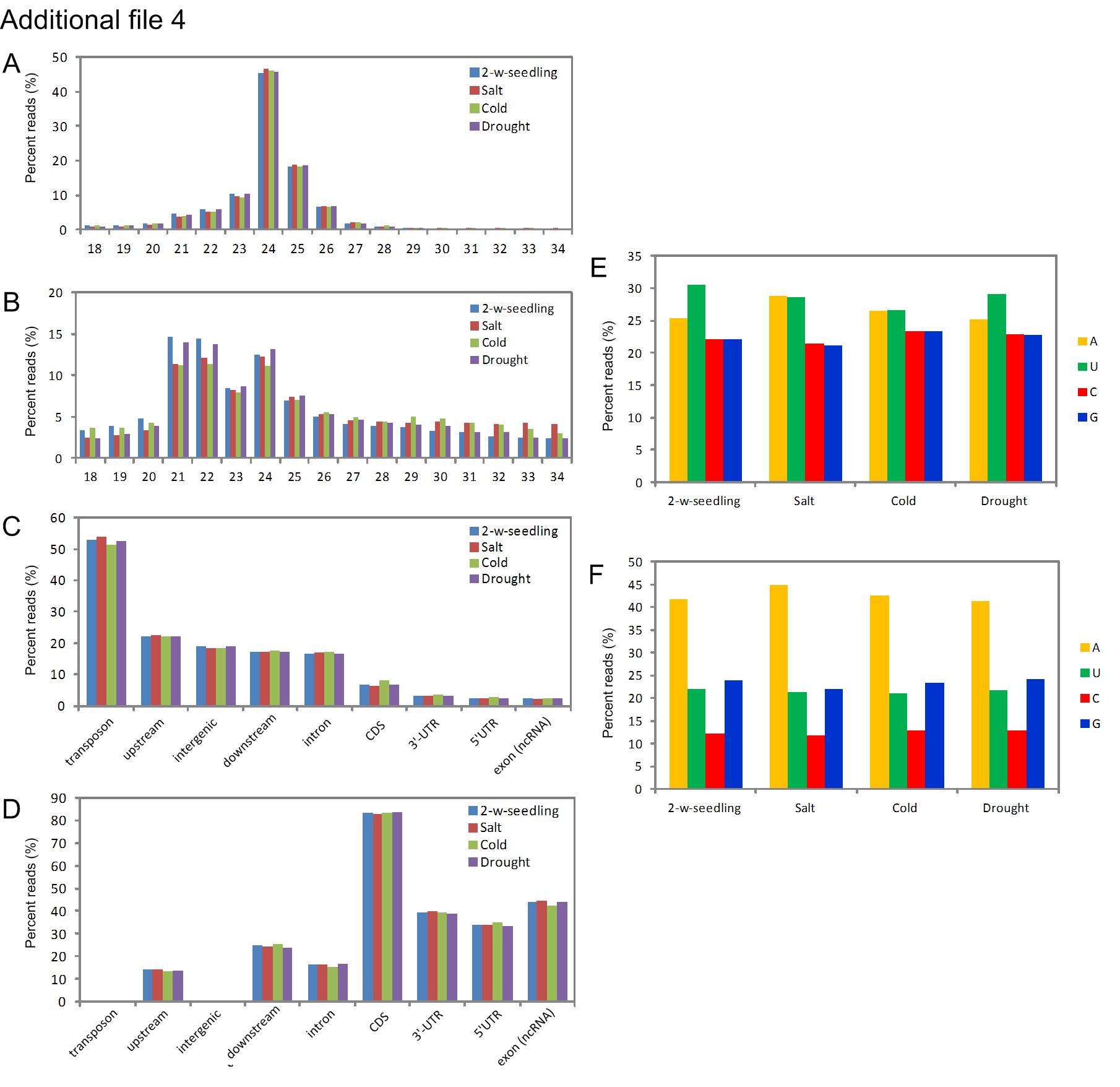

Supplement: Additional file 4 — Overview of small RNAs in rice. (A) Distribution of the lengths of all unique small RNAs generated by high-throughput sequencing from rice under four different conditions. (B) Length distribution of nat-siRNAs. (C) Distribution of all unique small RNAs located in different sequence components. (D) Distribution of nat-siRNAs located in different sequence components. The blue, red, green and purple bars represent small RNAs or nat-siRNAs from normal, salt stressed, cold stressed and drought stressed conditions, respectively (A-D). (E) First-nucleotide distribution of all unique small RNAs under four different conditions. (F) First-nucleotide distribution of nat-siRNAs under four different conditions. [file 1471-2164-13-721-S4.jpeg]

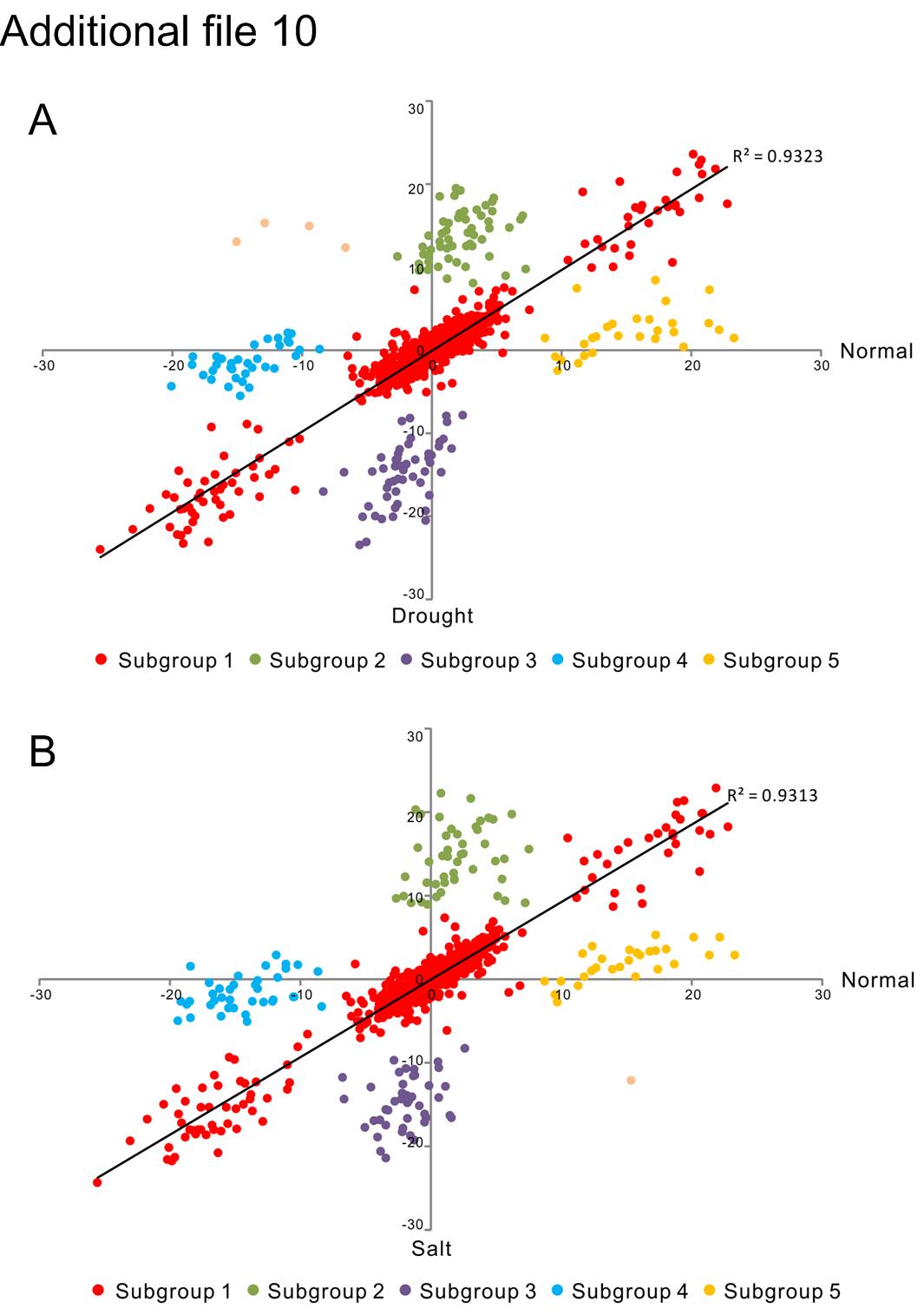

Supplement: Additional file 10 — Scatter plots of expression of 1072cis-NATs. The two scatter plots compare transcripts expression ratio trends of 1072 co-expressed cis-NAT pairs between normal and drought-stress conditions (A), and between normal and salt-stress conditions (B). The results show five subgroups: red, green, purple, blue and orange spots represent subgroups 1-5, respectively. [file 1471-2164-13-721-S10.jpeg]
