# Supplementary material for: Strand-specific RNA-seq reveals widespread occurrence of novel cis-natural antisense transcripts in rice
Source: BMC Genomics. 2012 Dec 22;13:721. doi: 10.1186/1471-2164-13-721 (PMC3549290; doi:10.1186/1471-2164-13-721)
Supplement: Additional file 13 — Statistics of 209 networks formed bycis-NAT groups. The numbers of one-to-two cis-NAT groups with expression evidence under normal, salt, cold and drought conditions are shown. These one-to-two cis-NAT groups were divided into five classes according to their cis-NATs types. Here, ‘All EXP’ indicates all cis-NAT groups with expression evidence (FPKM > 0) of both sense and antisense transcript, and with nat-siRNAs (number of small RNAs > 1) in overlapping region as well. ‘Either EXP’ indicates either of cis-NAT groups with expression evidence of both sense and antisense transcript, and with nat-siRNAs in the overlapping region as well. [file 1471-2164-13-721-S13.docx]

**Additional file 13.** Statistics of 209 networks formed by cis-NAT groups (1-to-2 type) with expression evidence.

| Type of cis-NAT groups | Number of groups | sd^a^ | | ST^b^ | | CD^c^ | | DT^d^ | | Co- | |
| --- | --- | --- | --- | --- | --- | --- | --- | --- | --- | --- | --- |
|  |  | All EXP^e^ | Either EXP^f^ | All EXP | Either EXP | All EXP | Either EXP | All EXP | Either EXP | All EXP | Either EXP |
| Convergent & divergent | 46 | 16 (34.7%) | 16 (34.7%) | 8 (17.4%) | 21 (45.7%) | 10 (21.7%) | 16 (34.8%) | 10 (21.7%) | 20 (43.5%) | 4 (8.7%) | 13 (28.3%) |
| Enclosed & enclosed | 74 | 18 (24.3%) | 36 (48.6%) | 22 (29.7%) | 31 (41.9%) | 23 (31.1%) | 30 (40.5%) | 22 (29.7%) | 30 (40.5%) | 6 (8.1%) | 30 (40.5%) |
| Divergent & enclosed | 40 | 12 (30.0%) | 15 (37.5%) | 8 (20.0%) | 16 (40.0%) | 12 (30.%) | 16 (40.0%) | 12 (30.%) | 16 (40.0%) | 2 (5.0%) | 19 (47.5%) |
| Convergent & enclosed | 48 | 15 (31.3%) | 17 (35.4%) | 15 (31.3%) | 14 (29.2%) | 14 (29.2%) | 14 (29.2%) | 14 (29.2%) | 14 (29.2%) | 6 (12.5%) | 19 (39.9%) |
| Convergent & convergent | 1 | 1 | - | 1 | - | 1 | - | 1 | - | 1 | - |

^a^ rice 14-day-old-seedling.

^b^ rice 14-day-old-seedling treated with 200 mM NaCl.

^c^ rice 14-day-old-seedling grown under cold stress at 4°C for 24 h in dark.

^d^ rice 14-day-old-seedling treated with 20% PEG-6000.

^e^ all of cis-NAT groups with expression evidence of both sense and antisense transcript, and with nat-siRNAs in overlapping region as well

^f^ either of cis-NAT groups with expression evidence of both sense and antisense transcript, and with nat-siRNAs in overlapping region as well
