# Supplementary material for: Strand-specific RNA-seq reveals widespread occurrence of novel cis-natural antisense transcripts in rice
Source: BMC Genomics. 2012 Dec 22;13:721. doi: 10.1186/1471-2164-13-721 (PMC3549290; doi:10.1186/1471-2164-13-721)
Supplement: Additional file 14 — Primers designed for real-time RT-PCR and Northern Blots in this research. (DOCX 13 kb) [file 1471-2164-13-721-S14.docx]

**Additional file 14.** Primers designed for real-time RT-PCR and Northern Blot in this research.

|  | cis-NATs | primer |
| --- | --- | --- |
| Northern Blot | Os05t0500000-00_Os05t0500101-01-Left | CAGGAGTAGTGCATCGCTCA |
|  | Os05t0500000-00_Os05t0500101-01-Right | GGGATTGCTCGTCAGAGATT |

|  | cis-NATs | primer |
| --- | --- | --- |
| real time RT-PCR | Os09t0482800-02-Left | ATAGCGGCAACTCGTTCTCA |
|  | Os09t0482800-02-Right | AAATCTCCACGCTCATCTCG |
|  | CUFF.14823.1-Left | TGTCAGCGTTTAGCTCCACTT |
|  | CUFF.14823.1-Right | TGCGTACTGTCCTCTTGCTG |
